# Efficacy of rocket and mustard oils and their nano-emulsions as alternatives to chemical herbicides for controlling weeds associated faba bean

**DOI:** 10.1038/s41598-025-29915-1

**Published:** 2025-12-09

**Authors:** Mona A. El-Wakeel, Faten S. A. Zaki

**Affiliations:** Botany Department, Agriculture and Biological Institute, National Research Centre, 33 El Bohouth st, Dokki, P.O. 12622, Giza, Egypt

**Keywords:** Musrard oil, Rocket oil, Nano-emulsion, Weed control, Physiological response and faba bean, Biochemistry, Physiology, Plant sciences, Systems biology, Environmental sciences

## Abstract

Weeds are one of the most well-known biotic environmental stresses in crop ecosystems. They negatively affect plant growth by competing for essential natural resources, leading to adverse impacts on physiological and biochemical processes. Chemical herbicides are also considered as abiotic stress and negatively affect on human and animal health as well as the surrounding environment. So, we shed light on natural products as rocket salad and mustard oils and their nano-emulsions as alternatives to chemical herbicides. Two pot experiments in greenhouse along the winter seasons of 2021/2022 and 2022/2023. Treatments involved foliar spray of rocket and mustard oils and their nano-emulsions at successive concentrations (2.5, 5.0 and 7.5%). Healthy uninfected faba bean plants and unweeded control treatments were applied for comparison. Recorded results revealed that all applied weed control treatments significantly decreased canary grass and cheeseweed growth parameters. Mustard oil was more efficient than rocket oil in suppressing both investigated weeds. This recorded reduction was directly proportional to concentration. Nano-emulsions applications recorded better results than pure oils (without nano-emulsions). Hence, Mustard oil nano-emulsions at 7.5% recorded notified inhibition for both weeds. The reduction of weeds’ biotic stress is positively reflected in turn on faba bean plants. In this respect, rocket oil showed a stimulatory effect on faba bean plants higher than mustard oil. Meanly, rocket oil at 7.5% scored the highest growth traits and photosynthetic pigment at both growth ages. Rocket and mustard oils at the highest concentration (7.5%) progressed on healthy faba bean in scoring the high yield traits and seed quality as compared to the unweeded control. GC-mass fractionation of both oils identified eight fatty acids, namely: palmitic, palmitoleic, stearic, oleic, linoleic, linolenic, behenic and erucic fatty acids. Inhibitory response of weeds and stimulatory response of faba bean plants may be attributed to these phenolic acids. Rocket and mustard oils and their nano-emulsions achieved our hypothesis in having herbicidal properties as they have a high phytotoxic effect on growth and physiological processes of the weeds.

## Introduction

 Faba bean (*Vicia faba* L.) is a winter grain legume that serves as a valuable component of the human diet in Egypt, providing a rich source of protein (18–32%), carbohydrates (55–63%), minerals (2–3.5%), fat (0.5–5.6%), phosphorus, iron, calcium, vitamins and antioxidant compounds^1,2^. The seeds of faba beans are particularly rich in the essential amino acids lysine and arginine, making them an excellent protein source for feeding farm animals. Faba beans have the ability to fix atmospheric nitrogen (N), which enriches the soil with N and organic matter, improving water use efficiency in the crop system. This makes faba beans a crucial crop for use in crop rotation, especially in dry regions^3^. The use of faba beans in crop rotation also enhances soil humus^1^. Due to the pyrimidine glycosides vicine and convicine (v-c), which are stored in the cotyledons of the majority of faba beans at 1% of dry matter, their consumption have historically been limited^4^.

Weeds have a detrimental impact on faba bean yield and quality. In Egypt, the presence of canary grass weed has led to faba bean crop losses exceeding 50%^5^. Weeds, as a biotic stress factor, pose a significant challenge in agricultural systems. They diminish crop production by competing with crops for essential resources such as space, light, air, nutrients, and other growth-supporting factors^6^. Weeds can disrupt physiological and biochemical processes crucial for plant health, including water uptake, photosynthesis, relative water content, electrolyte leakage, and antioxidant levels associated with oxidative stress and the generation of reactive oxygen species^7^. Additionally, weeds can act as hosts for pests and diseases, contributing significantly to agricultural crop losses, with weeds alone accounting for the highest percentage (34%) of agricultural losses^8^. Currently, synthetic herbicides are widely used for weed management due to the limitations and high costs associated with manual or mechanical approaches. However, the excessive use of synthetic herbicides has led to various environmental concerns, such as the development of herbicide-resistant weeds and adverse effects on human health, animal well-being, crop quality, and the surrounding ecosystem^9^. Consequently, there is a growing interest in organic products among consumers, businesses, and researchers, emphasizing the need for high-yield organic crops that are free from chemical treatments. Bioactive natural products are increasingly recognized as eco-friendly tools for safe and sustainable weed management. These natural compounds can be harnessed to develop organic herbicides based on the structural properties of natural phytotoxins, offering a wide range of biological activities^10^. The utilization of natural products such as essential oils, fixed oils, plant extracts, and other natural by-products is emerging as a viable alternative to conventional chemical herbicides. These natural alternatives are known for their rapid degradation in the environment, ensuring minimal impact on ecosystems. Natural by-products, in particular, are fast-degrading bioactive compounds in the soil that do not leach into groundwater, making them environmentally safe options for natural weed control^11^.

The Brassicaceae family, comprising approximately 375 genera and 3200 species, includes rocket salad (*Eruca sativa*) and white mustard (*Sinapis alba* L.), which are still cultivated in Atlantic and Mediterranean regions^12,13^. Rocket fixed oil exhibits high variability in fatty acid content, consisting of saturated fatty acids such as behenic (22:0), stearic (18:0), and palmitic (16:0) acids, as well as unsaturated fatty acids like erucic (22:1), ecosenoic (20:1), linolenic (18:3), linoleic (18:2), oleic (18:1) and palmitoleic (16:1) acids^14,15^. Notably, rocket oil contains glucosinolate methylsulphinylbutyl isothiocyanate, which induces enzyme activity^16^. White mustard, an important annual oilseed crop globally, is characterized by sulphur-containing secondary metabolites, namely glucosinolates, responsible for its bitter taste. The biological activity of glucosinolates can be attributed to their hydrolytic products^17^. Mustard seeds are a rich source of bioactive components, including polyunsaturated fatty acids and antioxidants (carotenoids, phenolic compounds, and tocopherols)^18^. The most abundant fatty acids in seeds are palmitic acid (16:0), stearic acid (18:1), oleic acid (18:0), linoleic acid (18:2), and linolenic acid (18:3), with very long chain fatty acids also present in triacylglycerols in significant amounts^19^. Das et al.^20^ detected an abundant amount of glucosinolates and omega-3 fatty acids in mustard seeds. Nano-emulsion technology is often employed to encapsulate bioactive substances in aqueous solutions with extremely small sizes, ranging from 20 to 200 nm. Nano-emulsions (NEs) consist of three main parts: oil, surfactant, and water, with the two immiscible phases (oil and water) separated by interfacial tension induced by surfactants^21^. In oil/water NEs, oil droplets are distributed in the continuous phase (water), transferring lipophilic active compounds. Before emulsions are formed, the lipophilic active substances are typically solubilized in the oil phase. The stability of NEs is a significant advantage for product encapsulation and is essential for maintaining the product’s surface oil content^22^. High-energy emulsification techniques, including high-speed homogenizers, are used to create NEs, which create the smallest droplet sizes while supplying the available energy in the shortest amount of time^23^. The use of natural nano-herbicides created from natural fixed oils would be a promising tool for controlling weeds as an alternative to synthetic chemical herbicides.

The hypothesis of this investigation was to evaluate the efficacy of applying rocket and mustard oils, as well as their formulated nano-emulsions (NEs), as natural organic bioherbicides for controlling weeds associated with faba bean plants. Therefore, the present study aimed to evaluate the herbicidal potential of selected natural products of rocket and mustard oils as well as their dormulated nano-emulsions (NEs) at different concentrations against major weeds of faba bean i.e., canary grass as a grassy weed and cheeseweed as a broad-leaved weed. Additionally, it revealed their impact on crop growth and development. This dual focus is intended to identify sustainable alternatives to synthetic chemical herbicides that can contribute to environmentally friendly weed management in faba bean cultivation.

## Materials and methods

### Materials

Faba bean (*Vicia faba* L. cv. Misr3), rocket salad *(Eruca sativa*), mustard (*Sinapis alba*), canary grass (*Phalaris minor*) and cheeseweed (*Malva parviflora*) seeds were purchased from The Agriculture Research Centre, Giza, Egypt. The nonionic surfactants (Tween 20 and Tween 80) and anionic surfactants (Sodium Dodecyl Sulfate) were purchased from Sigma-Aldrich Co. (St. Louis, MO, USA). All chemicals are of analytical grade and Milli-Q water (Millipore, USA) was used throughout sample preparations.

### Oil extraction

Rocket salad and mustard seeds were crushed using a household mill (Braun, Germany). Oil content of crushed seeds was extracted as described by the method mentioned in the A.O.A.C^24^ using petroleum ether (40–60 °C) in soxhlet apparatus.

### Determination of fatty acid composition

Fatty acid methyl esters of the oil were prepared by refluxing the sample (90 min) with a mixture of methanol: benzene: concentrated sulfuric acid (20: 10: 1) in a round bottom flask on a water bath according to Harborne^25^. The fatty acid compositions were performed with Hewlett Packard HP 6890 Gas chromatography (GC) with flame ionization detector (FID) on a split injector.

### Preparation of nano-emulsions (NEs)

High speed homogenization method was used according to Metwally et al.^26^ with some modifications to formulate oil-in- water NEs of two fixed oils: In this method, two phases were prepared: an oil phase and an aqueous phase. Both phases were combined to formulate the oil NEs. Rocket salad and mustard oils at 2.5%, 5.0% and 7.5% were used as the oil phase, whereas the aqueous phase consisted of Tween 20 and Tween 80 (1:1) as a nonionic surfactant at 2% and SDS at 4% as an anionic surfactant and deionized water. The oil phase was added slowly drop by drop to the aqueous phase at 25 °C with a high speed homogenization (20000 rpm) for 30 min. Then, the resulted NEs were subjected to ice to reduce the resulting heat and the produced NEs were kept at 4 °C for further analysis and bioassay.

### Characterization of NEs

#### NEs foam test

The test was conducted in accordance with WHO^27^ guidelines, using 75–80 ml of distilled water in a 250–ml beaker that had been heated to 30–31 °C. The NEs were then continuously added while being stirred with a glass rod at a speed of roughly 4 revolutions per second. The beaker was filled to a total of 100 ml with distilled water while being stirred. The liquid was then transferred into a 100-ml graduated cylinder that was clean, dry and airtight. The NEs were added and the emulsion was stirred for 3 min before being put into the 100-ml cylinder. After one hour at 30–31 °C, the cylinder was checked for creaming or phase separation. A NEs foam test was also performed to evaluate the levels of foam formed on the emulsion surface in the cylinder after 5 min.

#### NEs stability test

Stability test was conducted on the NEs by observing the droplet size of the NEs every 7 for 28 days. Throughout this test, the NEs were held at room temperature. Three sample drops were diluted into 10 mL of water, using a similar procedure to the one previously described, before droplet size analysis was performed. The creaming process was also seen with the naked eye to determine if it had taken place or not.

#### Particle size, polydispersity index and zeta potential analysis

Particle size of the NEs was measured by dynamic light scattering (DLS) method (Malvern Nano ZS, Malvern Panalytical Ltd., Malvern, Worcestershire, UK) at a measurement angle of 90° using a 633 nm laser with average of 13 runs for each measurement. To avoid any multi-scattering phenomenon, the sample was diluted before analysis by adding 3 drops of each sample to 10 mL of water and sonicating for 10 min. The sample was tested at room temperature and the average of three measurements was used to determine the findings. The software’s correlation function was used to determine the intensity-based average diameter (Z-average diameter).

#### Transmission electron microscope analysis (TEM)

The Morphology of oil NEs was pictured by TEM. TEM was used to analyze the droplet shape in the oil NEs^28^. After being applied on an ultra-thin TEM grid for 10 s, oil NEs (10 µl) were stained for 20 s with uranyl acetate negative stain solution. The TEM grids were then cleaned three times by contacting them with a drop of deionized water’s surface. The TEM grids were then dried at 25 °C over night. Then, using a 12,000x magnification and a 200 kV working acceleration voltage, the oil droplet shape was examined.

### Experimental procedure

Two pot experiments were carried out along two successive winter seasons (2021/2022) and (2022/2023) in the greenhouse of The National Research Centre, Dokki, Giza, Egypt. This study was applied to assess the impact of foliar spraying of rocket and mustard oils and their NEs at successive concentrations (2.5, 5.0 and 7.5%) on faba bean plants and associated weeds i.e., canary grass as a grassy weed and cheeseweed as a broad-leaved weed. In order to ensure consistency, faba bean seeds of the same size and color were chosen. Faba bean seeds (8seeds/pot) were sown at 3 cm from the soil surface in pots (30 cm diameter) filled with 10 kg of soil of a loamy mixture with clean sand soil at a ratio 1:1 w/w. All treatments, except the healthy control were infected with the seeds of canary grass and cheese weed. At the early vegetative stage (12–15 BBCH); approximately two weeks later, faba bean plants were thinned to 5plants/pot. Faba bean plants and two associated weeds were foliar sprayed twice at 21 and 30 days after sowing (DAS) with rocket and mustard oils and their NEs. At the first spray (21 DAS), faba bean plants were at 13–14 BBCH, canary grass at 12–13 BBCH and cheeseweed at 13–14 BBCH. At the second spray (30 DAS), faba bean plants reached 16–17 BBCH, canary grass 14–15 BBCH and cheeseweed 15–16 BBCH. Each pot received NPK at the recommended dose.

Pots were arranged in a randomized complete block design with nine replicates. The experiment consisted of 14 treatments as follows: two control treatments of healthy faba bean only and unweeded faba bean plants. Three treatments were sprayed with rocket oil and three treatments were sprayed with its NE at (2.5, 5.0 and 7.5%). The remained six treatments, three of them were sprayed with mustard oil and three treatments were sprayed with its NE at (2.5, 5.0 and 7.5%). Both oils and their NEs treatments at successive concentrations were applied using a hand-held sprayer (e.g., [Model/Brand, Manufacturer, Country]) equipped with a flat-fan nozzle (e.g., TeeJet XR 11002) at a working pressure of 200–250 kPa to ensure uniform coverage. The spray volume was adjusted to the recommended field application rate of 200 L feddan^–1^ (≈ 476 L ha^–1^). Considering the pot surface area (30 cm diameter ≈ 0.0707 m^2^), the equivalent spray volume corresponded to approximately 3.4 mL solution per pot.

### Sampling and data recording

(A) Weeds.

Weeds were hand pulled from pots at 45 and70 days after sowing (DAS), when canary grass was and cheeseweed was at 16–17 BBCH and 20–22 BBCH Fresh weight (FW) was separately recorded to canary grass and cheese weed. Collected separated weeds were dried in oven at 70 °C for 72 h till it reached to constant weight and dry weight (DW) was recorded and % reduction of each weed biomass was recorded.

(B) Faba bean plants.

### Faba bean growth

“The first sampling was conducted at the vegetative growth stage (30–39 BBCH; approximately 45 DAS), while the second sampling was performed at the flowering stage (60–69 BBCH; approximately 70 DAS) to evaluate morphological and physiological characteristics.” The recorded characteristics included: shoot height (cm), number of leaves/plant, number of branches/plant, fresh and dry weight of plant (g) as well as leaf area (cm^2^/plant).

### Estimation of photosynthetic pigments

Photosynthetic pigments (Chlorophyll a & b and carotenoids) were extracted from the upper fresh fourth leaf in each plant sample at growth stage (30–39 BBCH) and flowering stage (60–69 BBCH)using N, N-dimethylformamide according to method described by Moran^29^. The filtrate was measured against blank at three wave lengths (663.8, 646.8 and 470 nm) by JASCO V-750 spectrophotometer. Values were used to quantify chlorophyll contents as mg/g fresh weight of faba bean leaves.

### Yield criteria

The third sampling was conducted at the harvest maturity stage (89 BBCH; approximately 130 DAS). Three plants from faba bean were taken from each replicate to determine: number of pods/ plant, weight of pods/ plant (g), number of seeds/plant, weight of seeds/plant (g), weight of 100 seeds (g) and weight of straw (g).

### Quality of faba bean seeds

#### Chemical composition

Seeds from each treatment were collected, cleaned and crushed to tiny powder using a coffee grinder and prepared for analysis.

#### Total nitrogen and protein (TN &TP)

Total nitrogen (N %) content was determined by the Micro- Kjeldahl method described by A.O. A. C.^30^. Total protein content (TP %) was calculated by multiplying the value of total nitrogen by 6.25.

$${\text{TP }}\left( \% \right)\,=\,{\text{TN}}\% \times {\text{6}}.{\text{25}}$$

#### Total soluble sugar (TSS)

The phenol-sulfuric acid method was used for the determination of total soluble sugar in the faba bean seeds^31^. The absorbance was read with JASCO V-750 spectrophotometer at 490 nm based on standard curve of glucose against blank and then expressed as mg g^− 1^ DW.

#### Total free amino acid (TFAA)

Free amino acid content was determined with the ninhydrin reagent method^32^. The absorbance was read at 570 nm in a UV- Spectrophotometer (JASCO V750). Glutamic acid was used as a standard amino acid to prepare a calibration curve.

#### Total phenolic compounds (TPC)

TPC content of faba bean seeds was determined using Folin and Ciocalteu phenol reagent according to the method defined by Elzaawely and Tawata^33^. The absorbance was measured at 765 nm with a JASCO V-750 spectrophotometer. TPC was calculated from a standard curve of gallic acid and expressed as mg gallic acid equivalent /g DW.

#### Total flavonoid compounds (TFC)

TFC of each extract was determined using the aluminum chloride technique modified by Chang et al.^34^. The absorbance was measured at 415 nm against the blank using a JASCO V-750 spectrophotometer. TFC was calculated from a standard curve of quercetin and expressed as mg quercetin equivalent/g DW.

### Statistical analysis

The recorded data were submitted to homogeneity test before to analysis of variance (ANOVA). ANOVA was performed on the data for each season in accordance with Casella^35^ using the Costat software, Version 6.303, 2004. The means of applied treatments were separated using Tukey’s HSD test at the 0.05 level of probability.

## Results

### Fatty acid profiles of rocket and mustard seed’s oil

**Fig. 1:**
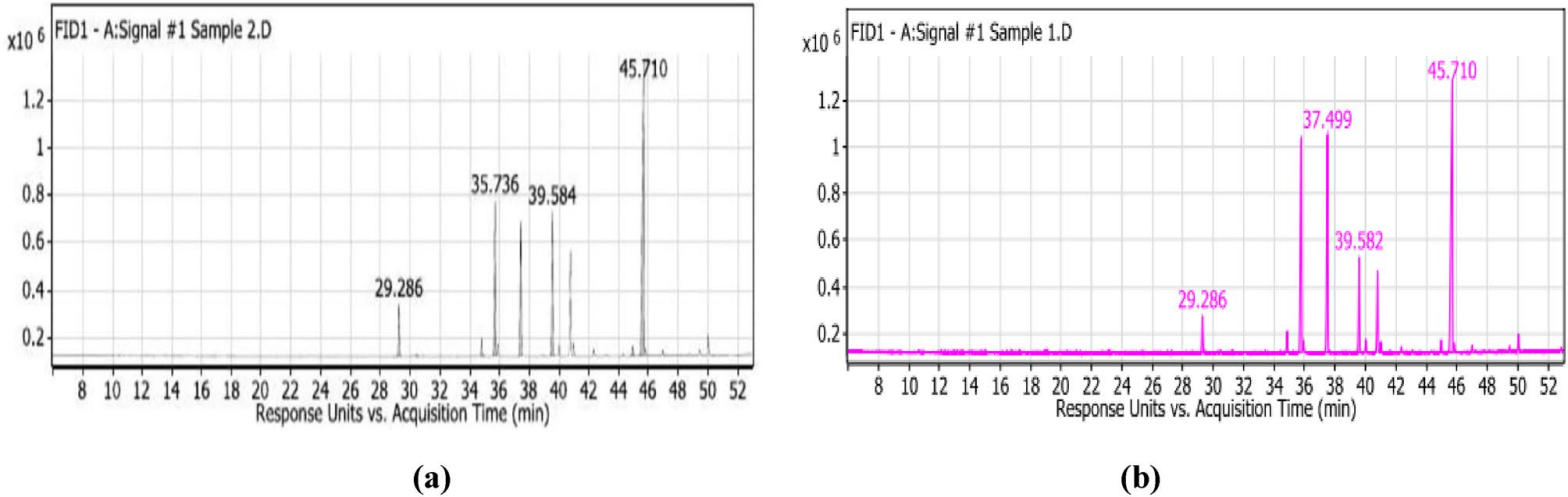
Gc-mass fractionation of fatty acids (a) Rocket seed oil. (b) Mustard seed oil.

The extracted oils were evaluated for their fatty acid (FA) compositions. It could be realized from the tabulated results (Table 1) that both rocket and mustard seed oils exhibit nearly equal total FA percentage and similar FA profiles but their quantities are different. Eight fatty acids namely: palmitic acid (C16:0), Palmitoleic acid (C16:1), stearic acid (C18:0), oleic acid (C18:1), linoleic acid (C18:2), linolenic acid (C18:3), behenic acid (C22:0) and erucic acid (C22:1) were identified (Fig. 1). Fatty acid analysis indicated a higher content of erucic acid in rocket and mustard oils being 47.63 and 39.72% of the total FAs, respectively. In addition, mustard oil has more oleic acid and linoleic acid than rocket oil being 23.09 and 22.18% of the total FAs, respectively.

**Table 1:** Fatty acid compositions (%) of rocket and mustard seed oils.

Fatty acids composition (%)	Rocket seed oil	Mustard seed oil
Palmitic acid (C16:0)	4.98	3.21
Palmitoleic acid (C16:1)	0.22	0.13
Stearic acid (C18:0)	1.70	1.89
Oleic acid (C18:1)	16.17	23.09
Linoleic acid (C18:2)	13.78	22.18
Linolenic acid (C18:3)	14.53	8.72
Behenic acid (C22:0)	0.97	1.06
Erucic acid (C22:1)	47.63	39.72
Saturated fatty acids (USFA)	7.65	6.16
Unsaturated fatty acids	92.33	93.84
Total fatty acids %	99.98	100.00

### Characterization of oil NEs

#### NEs foam test

The foam test was passed by all formulations for both types of emulsified oil nano-emulsions. After 1 min, the foam height for both types of oil-based nano-emulsions was less than 1 ml.

#### NEs stability test

After one month of stability testing at room temperature, all formulations for both types of nano-emulsions of emulsified oil passed; all compositions were transparent pale yellow. After 30 days of storage, neither flocculation nor the separation of a creamy layer was seen. Phase separation, settling and agglomeration were not detected in any of the formula’s stability test findings after a month of storage at 50 °C. Finally, the results of the foam test and stability test provide guidance for the optimal nano-emulsion formulation and wise surfactant type and ratio selection.

#### Particle size, polydispersity index and zeta potential analysis

**Fig. 2:**
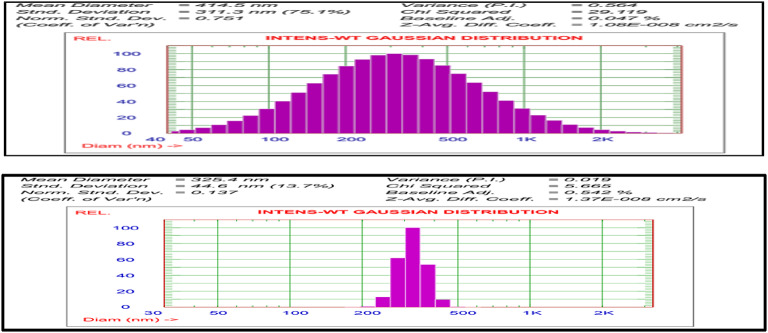
Particle size distribution of rocket and mustard oil NEs.

Particle size analysis was carried out to determine the stability of NE. To determine the stability of NE, the particle size distribution was performed. According to DLS measurements, the rocket and mustard NE had an average particle size of 414.5 nm and 325.4 nm, respectively (Fig. 2). The figure showed that 25% of the droplets had diameters less than 190.3 and 293.7 nm for rocket and mustard NE oils, respectively. One of the key parameters affecting the physicochemical and functional characteristics of NE, including stability, rheology and chemical reactivity, is the particle size analysis.

In order to examine the homogeneity of particle size, the polydispersity index (PdI) is calculated. The PdI value of the rocket and mustard NE was 0.56 and 0.01, respectively. A lower PdI value (0.3) denotes a highly stable mono dispersed NE, whereas a higher PdI value (> 0.7) defines a low degree of uniformity of NE with an extensive dispersion of particle. In the current study, the application of the high speed homogenizer resulted in a NE with a PdI value less than 0.3, indicating the successful synthesis of evenly sized stable NE.

An important element that determines a NE’s stability is its zeta potential. Due to the non-ionic Tween 80 surfactant and the negative charge of the nanoparticle droplets, the surface of NE was negatively charged and had an average zeta potential of about − 33.56 and − 29.88 mV for rocket and mustard NE, respectively according to DLS Zetasizer measurements. The existence of functional groups in the chemical components of rocket and mustard oils and the adsorption of negative ions onto the surfaces of oil droplets are both contributing factors to the creation of the negative charges in NE.

#### Transmission electron microscope (TEM)

**Fig. 3:**
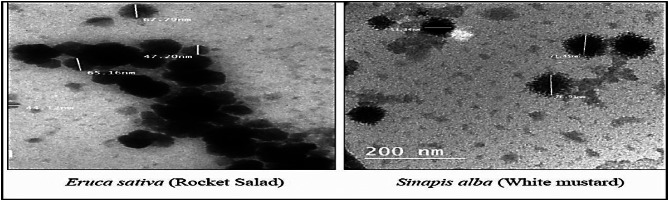
TEM image of rocket and mustard oil NEs.

Morphology, size and shape of rocket and mustard oil NEs were determined via TEM examination. A drop of diluted solution was applied to a copper grid that had been coated with carbon before being dried under a light to create the TEM grid. TEM pictures confirmed NEs particle size. Nano-emulsions had milky color and lower transmission percentage ranged from 47 to 65 for rocket oil and 53.3–76.9 for mustard oil. The particles had sphere-like shapes. Results determined by TEM analysis was less than that determined by DLS analysis Fig. 3.

### Efficiency of weed control

**Fig. 4:**
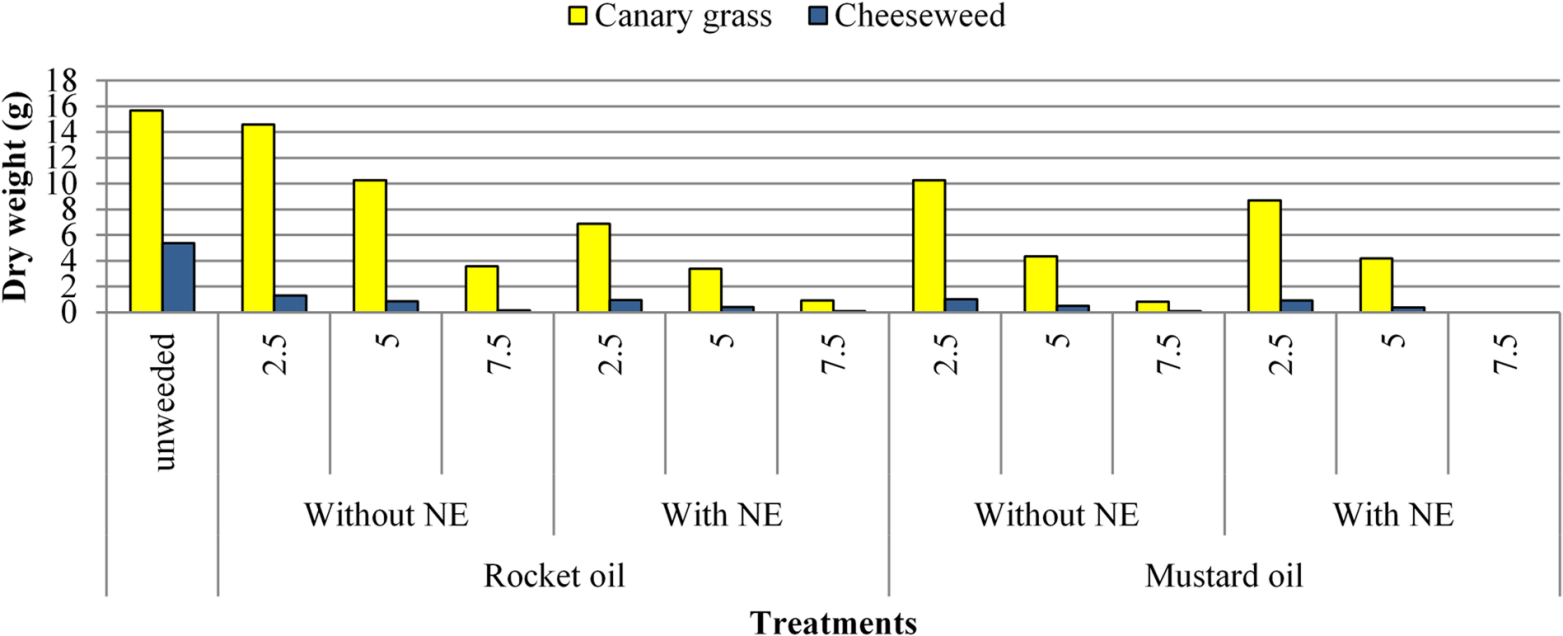
Canary grass and cheeseweed dry weight (g/pot) as affected by foliar application of rocket and mustard oils and their NEs at 70 DAS (mean of both seasons).

Tables 2 and 3 indicates that rocket and mustard oils and their NEs treatments at successive concentrations caused significant reductions in fresh and dry weight of canary grass and cheeseweed weeds at 45 days after sowing (DAS), when canary grass was at 16–17 BBCH and cheeseweed at 16–17 BBCH. At 70 DAS, canary grass reached 21–22 BBCH, while cheeseweed was at 20–21 BBCH. Recorded results presented revealed that each weed’s rate of reduction was directly attributed with increasing of the applied oils and their NEs concentrations. Thus, at both growth stages, NEs applications were more effective as inhibitory treatments than pure oils. It also observed that mustard oil and its NEs is more efficient in reducing fresh and dry weight of both weeds than rocket oil NEs (Fig. 4). Mustard oil NE at highest concentration scored the highest activity as natural herbicide and reached to complete elimination level of all weeds by scoring 100% inhibition. Pure mustard oil at 7.5% came in the second rank in scoring inhibition to both investigated weeds. Rocket NE at 7.5% followed these superior treatments in managing both weeds as compared to unweeded treatment.

**Table 2:** Effect of rocket and mustard oils and their NEs on Canary grass and Cheeseweed fresh and dry weights (g/pot) at 45 and 70 DAS (Combined analysis of the two seasons).

Treatments				At ((16–17 BBCH)					
				Canary grass			Cheeseweed		
				FW	DW	% Red.	FW	DW	% Red.
Control	Faba bean only			0.00a	0.00a	----	0.00a	0.00a	----
	Unweeded			21.14g	3.25i	0.00	5.49i	3.33j	0.00
Rocket oil	Without NE	%	2.5	9.63f	1.55 h	52.31	4.30h	2.11i	36.64
			5.0	5.65d	1.10hgf	66.15	2.11e	0.82e	75.38
			7.5	4.12c	0.67fedc	79.38	1.24c	0.32dc	90.39
	With NE		2.5	4.47dc	0.86gfe	73.54	2.29fe	1.27f	61.86
			5.0	2.20b	0.31dcba	90.46	1.13cb	0.26dcb	92.19
			7.5	1.95b	0.20cba	93.85	0.84b	0.19cb	94.29
Mustard Oil	Without NE	%	2.5	7.50e	1.21hg	62.77	3.02 g	1.92h	42.34
			5.0	5.05dc	0.97gfe	70.15	1.75d	0.66e	80.18
			7.5	1.11ba	0.10ba	96.92	0.15a	0.11ba	96.70
	With NE		2.5	4.32dc	0.76gfed	76.62	2.57f	1.48 g	55.56
			5.0	3.73c	0.55edcb	83.08	1.62d	0.41d	87.68
			7.5	0.00a	0.00a	100.00	0.00a	0.00a	100.00
P-value				**	**		**	**	
SE				± 0.26	± 0.10		± 0.06	± 0.03	

** Significative at a level of 1% of probability (*p* < .01), * Significative at a level of 5% of probability (0.01 = < *p* < .05), ns Non significative (p > = 0.05), DAS = days after sawing, NE = nano-emulsion, FW = fresh weight, DW = Dry weight, Red.= reduction and SE = standard error.

**Table 3:** Effect of rocket and mustard oils and their NEs on Canary grass and Cheeseweed fresh and dry weights (g/pot) at 45 and 70 DAS (Combined analysis of the two seasons).

Treatments				At (20–22 BBCH)					
				Canary grass			Cheeseweed		
				FW	DW	% Red.	FW	DW	% Red.
Control	Faba bean only			0.00a	0.00a	-----	0.00a	0.00a	-----
	Unweeded			59.96l	15.67f	0.00	8.39i	5.38 g	0.00
Rocket oil	Without NE	%	2.5	48.32j	14.58f	6.95	5.08hg	1.31f	75.65
			5.0	39.67h	10.27e	34.45	4.02gfe	0.87e	83.83
			7.5	16.50fe	3.59b	77.09	3.02ed	0.17cba	96.84
	With NE		2.5	39.81ih	6.88c	56.09	4.69hgf	0.95e	82.34
			5.0	12.94d	3.40b	78.30	2.22dc	0.42dc	92.19
			7.5	6.67c	0.91a	94.19	1.64cb	0.10cba	98.14
Mustard Oil	Without NE	%	2.5	42.28i	10.27e	34.45	5.42h	1.03fe	80.85
			5.0	18.55f	4.34b	72.31	3.71fe	0.51d	90.52
			7.5	3.52b	0.82a	94.76	0.85ba	0.08ba	98.51
	With NE		2.5	32.69 g	8.70d	44.49	4.82hg	0.93e	82.72
			5.0	14.34ed	4.19b	73.26	2.06dc	0.37dcb	93.13
			7.5	0.00	0.00a	100.00	0.00a	0.00a	100.00
P-value				**	**		**	**	
SE				± 0.50	± 0.25		± 0.21	± 0.06	

** Significative at a level of 1% of probability (*p* < .01), * Significative at a level of 5% of probability (0.01 = < *p* < .05), ns Non significative (p > = 0.05), DAS = days after sawing, NE = nano-emulsion, FW = fresh weight, DW = Dry weight and SE = standard error.

### Physiological parameters of faba bean

#### Growth traits

**Fig. 5:**
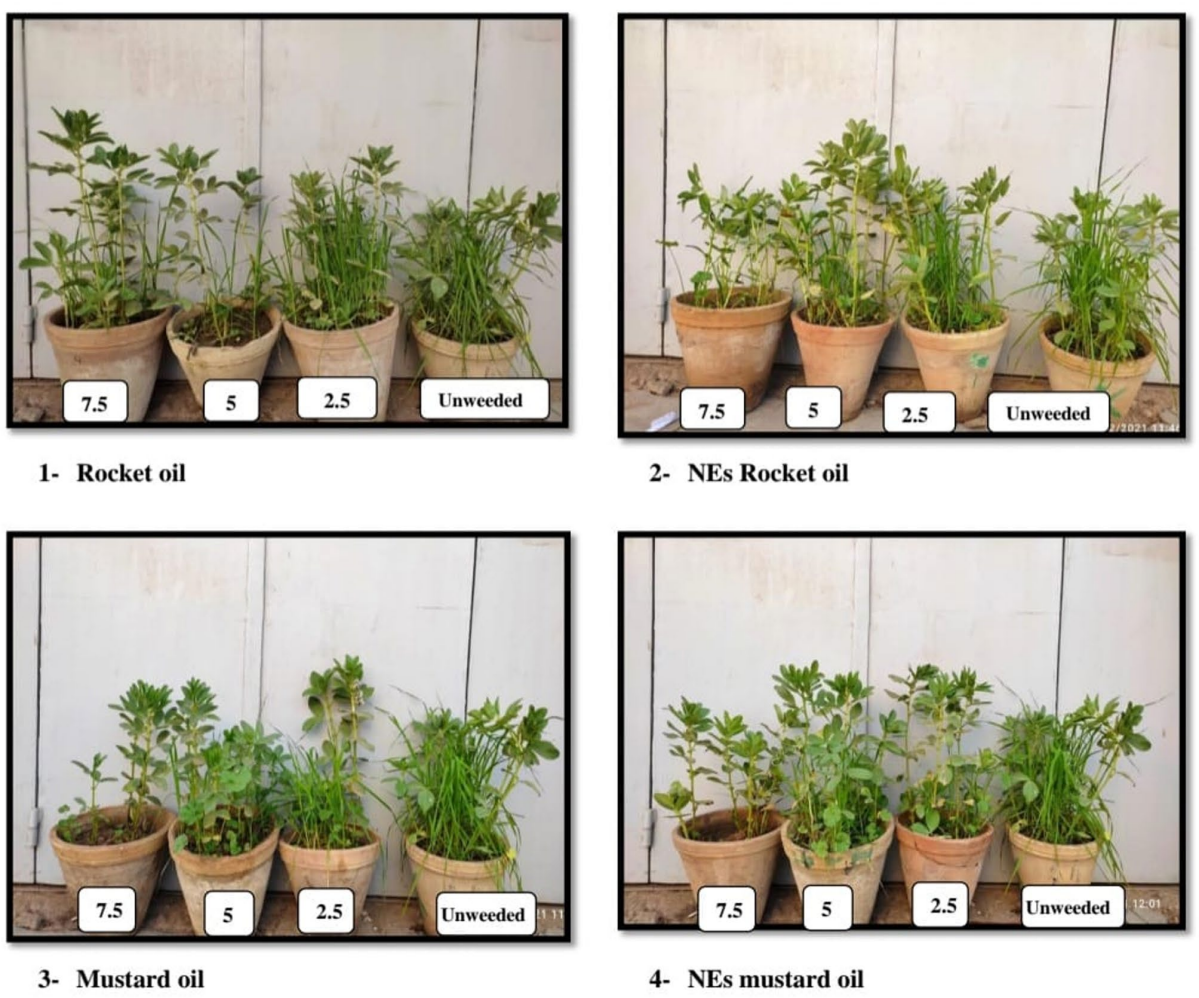
Growth of faba bean plants at the vegetative stage 30–39 BBCH (45 days after sowing) as a result of spraying faba bean and associated weeds with rocket and mustard oils and their NEs.

As presented in Table 4; Fig. 5, natural products rocket and mustard seed oils and their NEs at successive concentrations increased physiological parameters i.e., shoot height, number of leaves/plant, number of branches/plant, plant fresh weight, plant dry weight and leaf area at vegetative stage as compared to unweeded treatment. Generally, at vegetative growth 30–39 BBCH (45 DAS) pure rocket oil at highest concentration (7.5%) and mustard oil NE at lowest concentration (2.5%) were the distinctive practices for enhancing the aforementioned growth traits.

**Table 4:** Effect of rocket and mustard oils and their NEs on Faba bean vegetative growth parameters 30–39 BBCH at 45 DAS (Combined analysis of the two seasons).

Treatments						Vegetative stage (30–39 BBCH)			
				Shoot height (cm)	No. of leaves/ plant	No. of branches/ plant	Plant FW (g)	Plant DW (g)	LA (cm^2^/ plant)
Control	Faba bean only			41.00abc	13.00a	1.67a	12.33bcd	1.33cde	30.96d
	Unweeded			30.17d	10.50a	1.00a	7.91f	0.94e	16.94e
Rocket oil	Without NE	%	2.5	35.84cd	10.67a	1.00a	8.13f	0.96e	18.59e
			5.0	42.66abc	13.17a	1.83a	13.33abc	1.40bcd	34.88cd
			7.5	46.00a	14.00a	2.00a	15.98a	2.08a	58.14a
	With NE		2.5	36.50 cd	10.83a	1.25a	8.55ef	0.98e	19.42e
			5.0	44.17ab	13.50a	1.85a	13.35abc	1.70abc	36.36cd
			7.5	37.84bc	11.25a	1.50a	10.98cde	1.13de	31.41d
Mustard oil	Without NE	%	2.5	37.75bc	11.00a	1.34a	8.69ef	1.01de	20.21e
			5.0	44.63ab	13.42a	1.92a	13.44abc	1.76ab	42.93b
			7.5	39.00abc	12.50a	1.50a	11.55bcd	1.17de	35.50cd
	With NE		2.5	44.75ab	13.82a	2.00a	14.09ab	2.01a	54.56a
			5.0	42.83abc	13.00a	1.67a	11.67bcd	1.23de	39.65bc
			7.5	37.80bc	11.00a	1.42a	10.49def	1.09de	32.26d
P-value				**	ns	ns	**	**	**
SE				± 1.35	± 0.91	± 0.26	± 0.52	± 0.08	± 1.10

** Significative at a level of 1% of probability (*p* < .01), * Significative at a level of 5% of probability (0.01 = < *p* < .05), ns Non significative (p > = 0.05), NE = nano-emulsion, FW = fresh weight, DW = dry weight, LA = leaf area and SE = standard error.

Flowering stage 60–69 BBCH (70 DAS) as shown in Table 5, all the applied treatments significantly increased the investigated growth parameters except leaf area treated with pure rocket oil at 7.5% and pure mustard oil at 2.5% its NE at 7.5%. Additionally, it was observed that rocket oil was progressed on mustard oil in stimulation of faba bean growth parameters at highest concentration. Pure rocket oil at 7.5% and its NE at 5.0% scored the highest faba bean growth parameters. Healthy faba bean plants and pure mustard oil at 7.5% followed the aforementioned superior treatments. In both ages, it was observed that foliar spray of pure rocket oil significantly stimulated growth traits and this stimulation is directly proportional with its concentration. But, rocket NE oil and mustard oil either in pure or NE case also stimulated the growth traits but at highest concentration shoot height decreased to be lower than healthy control but not lower than unweeded treatment.

**Table 5:** Effect of rocket and mustard oils and their NEs on Faba bean flowering growth parameters at 60–69 BBCH (70 DAS) (Combined analysis of the two seasons).

Treatments						Flowering stage (60–69 BBCH)			
				Shoot height (cm)	No. of leaves/ plant	No. of branches/ plant	Plant FW (g/plant)	Plant DW (g/plant)	LA (cm2/plant)
Control	Faba bean only			56.25bc	21.59bc	2.67ab	51.15b	5.18abc	245.55ab
	Unweeded			36.50e	13.00f	1.33b	17.43f	1.78h	114.02b
Rocket oil	Without NE	%	2.5	51.42bcd	18.50de	2.00ab	39.42de	3.57defg	229.22ab
			5.0	53.17bcd	18.75cde	2.25ab	43.16cd	4.04bcde	232.76ab
			7.5	66.83a	28.67a	3.17a	59.32a	5.73a	359.02a
	With NE		2.5	50.00bcd	19.00bcd	1.84ab	39.22de	3.27defgh	213.58b
			5.0	58.25ab	22.00b	2.84ab	58.34a	5.49ab	246.48ab
			7.5	46.83cd	15.75ef	1.67ab	32.81e	2.13gh	123.90b
Mustard oil	Without NE	%	2.5	47.50cd	18.84cd	1.67ab	34.60e	2.53efgh	118.00b
			5.0	54.34bcd	19.59bcd	2.42ab	45.55bcd	3.97bcdef	239.18ab
			7.5	55.09bcd	20.67bcd	2.50ab	46.74bc	4.39abcd	241.61ab
	With NE		2.5	54.00bcd	19.75bcd	2.42ab	45.05bcd	3.23defg	237.40ab
			5.0	54.17bcd	18.75cde	2.42ab	45.42bcd	3.79cdef	232.94ab
			7.5	45.50de	15.00f	1.67ab	23.81f	2.42fgh	118.00b
P-value				**	**	*	**	**	**
SE				± 1.9	± 0.58	± 0.31	± 1.29	± 0.30	± 26.96

** Significative at a level of 1% of probability (*p* < .01), * Significative at a level of 5% of probability (0.01 = < *p* < .05), ns Non significative (p > = 0.05), NE = nano-emulsion, No. = number, FW = fresh weight, DW = dry weight, LA = leaf area and SE = standard error.

#### Photosynthetic pigment contents

Table 6 shows the changes in the contents of photosynthetic pigments (chlorophyll a, chlorophyll b, chlorophyll a + b and carotenoids) at different developmental stages of faba bean plants as a result of exogenous application of rocket and mustard oils and their NEs each at 2.5, 5.0 and 7.5%. It is evident from the results that untreated weed infection significantly reduced these photosynthetic pigments in faba bean leaves at 60–69 BBCH and 60–69 BBCH as compared to healthy control. Analyzed results indicated that rocket oil at 7.5% and NE mustard oil at 2.5% was most effective in increasing components of photosynthetic pigments at vegetative growth stage compared to unweeded control. However, rocket oil at 7.5% and healthy faba bean treatments scored the highest enhancement to the photosynthetic pigment at flowering stage. Hence, the superiority of rocket oil than healthy untreated control with significant difference between them is a good achievement in this study. Rocket oil with NE at 5.0%and pure mustard oil at 7.5% follow these superior treatments. Whereas, at flowering stage, only rocket oil either in pure phase at 7.5 or in NE emulsion phase at 5.0% progressed on healthy treatment as compared to unweeded treatment.

**Table 6:** Effect of rocket and mustard oils and their NEs on Faba bean photosynthetic pigment contents in leaves at the vegetative and flowering stages (Combined analysis of the two seasons).

Treatments				Vegetative stage (30–39 BBCH )				Flowering stage (60–69 BBCH )			
				Chl a	Chl b	Chl a + b	Cart	Chl a	Chl b	Chl a + b	Cart
Control	Faba bean only			0.50abc	0.15abc	0.65abc	0.15abc	1.50bc	0.50ab	2.00bc	0.47ab
	Unweeded			0.38d	0.12c	0.50d	0.10d	0.75g	0.20e	0.95g	0.21e
Rocket oil	Without NE	%	2.5	0.40 cd	0.13bc	0.53 cd	0.12 cd	1.16ef	0.33bcde	1.49def	0.36bcde
			5.0	0.45bcd	0.14abc	0.59bcd	0.16abc	1.21de	0.33bcde	1.54de	0.37bcde
			7.5	0.58a	0.16a	0.74a	0.17a	1.85a	0.59a	2.44a	0.55a
	With NE		2.5	0.40cd	0.13bc	0.53cd	0.13bcd	1.14ef	0.31bcde	1.45def	0.34bcde
			5.0	0.52abc	0.15ab	0.67ab	0.16abc	1.68ab	0.44abc	2.12ab	0.45abc
			7.5	0.48abcd	0.14abc	0.62abcd	0.15abc	0.87 g	0.24cde	1.11 fg	0.26de
Mustard Oil	Without NE	%	2.5	0.44bcd	0.14abc	0.58bcd	0.13abcd	0.95 fg	0.25cde	1.20efg	0.29cde
			5.0	0.47abcd	0.13bc	0.60bcd	0.16abc	1.33cde	0.38abcde	1.71cd	0.39abcd
			7.5	0.47ab	0.15ab	0.62abcd	0.15abc	1.39 cd	0.44abcd	1.83bcd	0.41abcd
	With NE		2.5	0.54abcd	0.16a	0.70ab	0.16abc	1.29de	0.34bcde	1.63 cd	0.39abcd
			5.0	0.49abcd	0.15ab	0.64abc	0.15abc	1.39 cd	0.38abcde	1.77bcd	0.39abcd
			7.5	0.45bcd	0.14abc	0.59bcd	0.15abc	0.81g	0.21de	1.02g	0.24de
P-value				**	**	**	**	**	**	**	**
SE				± 0.023	± 0.006	± 0.03	± 0.008	± 0.04	± 0.04	± 0.07	± 0.03

** Significative at a level of 1% of probability (*p* < .01), * Significative at a level of 5% of probability (0.01 = < *p* < .05), ns Non significative (p > = 0.05), NE = nano-emulsion and SE = standard error.

#### Yield traits

**Fig. 6:**
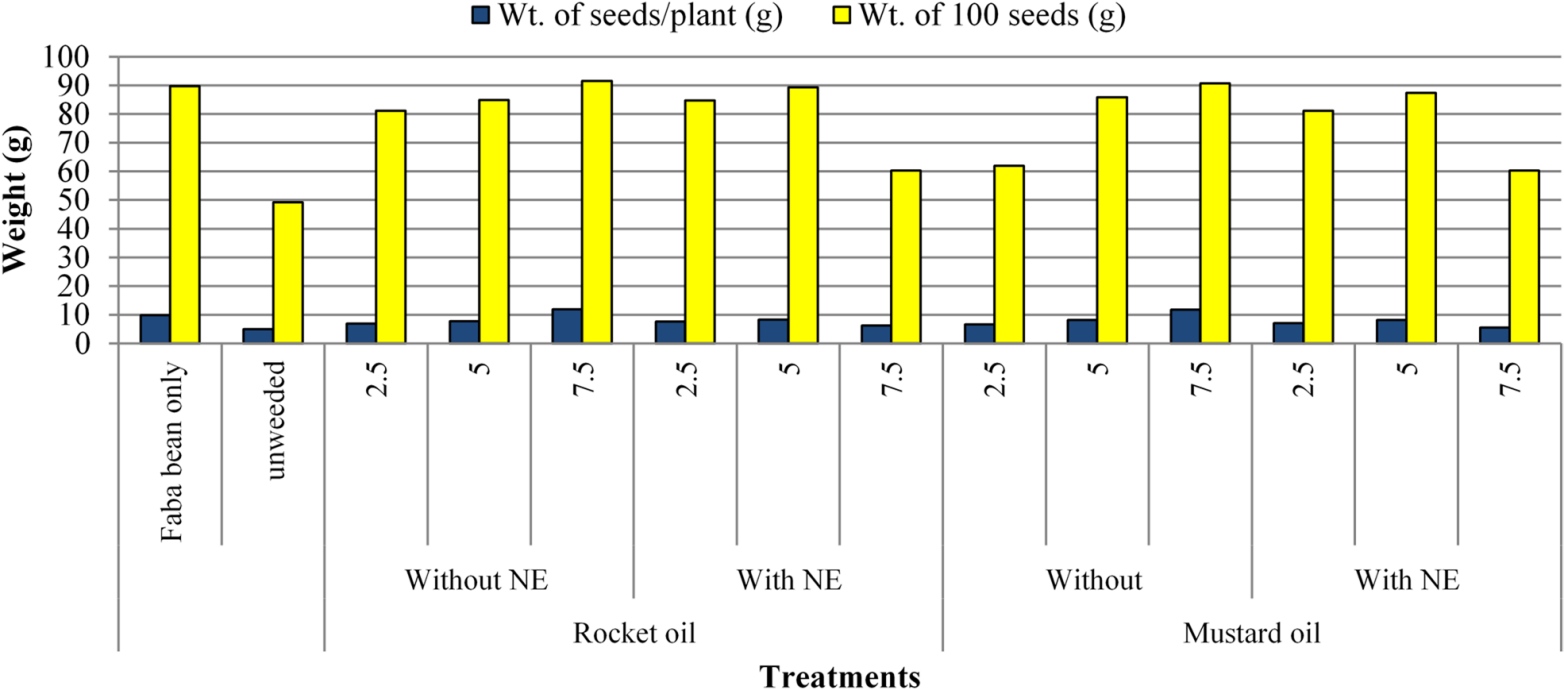
Response of weight of seeds and weight of 100 seeds /plant as a result of spraying of rocket and mustard oils as well as their NEs (mean of both seasons). Wt.= weight and NE = Nano-emulsion.

As shown from Table 7; Fig. 6, The different applied concentrations (2.5, 5.0 and 7.5%) of rocket and mustard seed oils and their NEs significantly promoted different yield traits (number of pods/plant, weight of pods (g/plant), number of seeds/plant, weight of seeds (g/plant), weight of 100 seeds (g) weight and of straw (g/plant) as compared with unweeded control plants. Rocket and mustard oils at highest concentration (7.5%) progressed on healthy faba bean in the aforementioned yield traits. This scored result achieved the hypothesis of the ability of using fixed oils as safe alternatives to enhance yield traits as equal as or higher than healthy control. Additionally, rocket and mustard oils NE at 5.0% came in the second rank in scoring high yield traits as compared to control.

**Table 7:** Response of Faba bean yield traits (60–69 BBCH) as a result of spraying of rocket and mustard oils as well as their NEs. (mean of both seasons).

Treatments				No. of pods/plant	Wt. of pods/plant (g)	No. of seeds/plant	Wt. of seeds/ plant (g)	Wt. of 100 seeds (g)	Wt. of straw/ plant (g)
Control	Faba bean only			6.17abc	12.53bc	18.15ab	9.27ab	89.80c	9.85b
	Unweeded			3.00h	3.63e	9.50 h	4.90h	49.20m	4.36l
Rocket oil	Without NE	%	2.5	3.84fgh	7.46d	13.50de	6.51ef	81.20i	6.84g
			5.0	4.50def	9.47e	14.17d	7.65cd	84.95 g	7.68e
			7.5	6.97a	15.72a	18.50a	9.76a	91.50a	11.83a
	With NE		2.5	4.30efg	8.04d	13.67de	7.66cd	84.70h	7.61e
			5.0	5.67bcd	11.50c	17.67b	8.53bc	89.30d	8.25c
			7.5	3.67fgh	6.98d	13.25e	6.04fg	60.29l	6.24i
Mustard oil	Without NE	%	2.5	3.71fgh	6.67d	11.67f	6.47ef	62.01j	6.66h
			5.0	4.30efg	9.59e	15.25gh	8.11cd	85.85f	8.09d
			7.5	6.67ab	14.23ab	18.25i	9.66a	90.65b	11.73a
	With NE		2.5	4.00fgh	7.92d	14.17d	7.35de	81.20i	7.04f
			5.0	5.42cde	11.28c	16.09c	8.35c	87.35e	8.19 cd
			7.5	3.25gh	6.62d	10.75 g	5.52gh	60.29 L	5.54j
p-value				**	**	**	**	**	**
SE				± 0.23	± 0.43	± 0.29	± 0.17	± 0.03	± 0.02

** Significative at a level of 1% of probability (*p* < .01), * Significative at a level of 5% of probability (0.01 = < *p* < .05), ns Non significative (p > = 0.05), NE = nano-emulsion, Wt = weight, No. = number and SE = standard error.

#### Seed quality

**Fig. 7:**
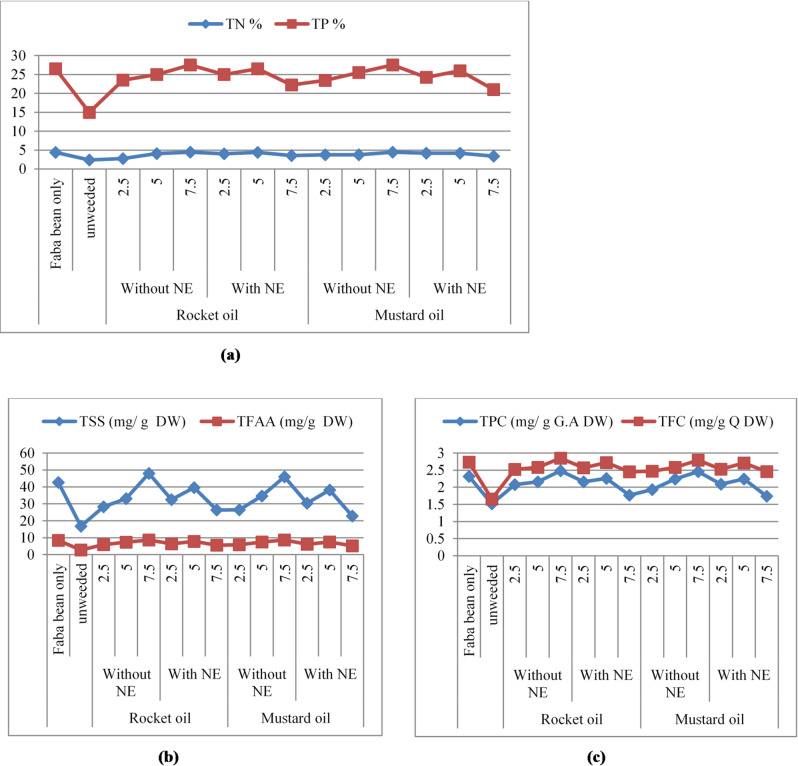
**(a)** Total nitrogen (TN) and total protein (TP). **(b)** Total soluble sugar (TSS) and total free amino acid (TFAA). **(c)** Total phenolic compounds (TPC) and total flavonoid contents (TFC) of faba bean seeds as a response for spraying with rocket and mustard oils and their NEs.

Results tabulated in Table 8 ensured that the investigated treatments at successive concentrations significantly increased the seed quality parameters i.e. total Nitrogen (TN), total protein content (TP), Total soluble sugar (TSS), total free amino acids (TFAA), Total phenolic compounds (TPC) and total flavonoid content (TFC) as compared to unweeded treatment. It was revealed that pure rocket and mustard oils at 7.5% scored the highest aforementioned faba bean seeds quality parameters (Fig. 7). These growth regulator efficient treatments followed with healthy control treatments and these findings are a good achievement from the present investigation.

**Table 8:** Chemical analysis of Faba bean seeds as a result of application of rocket and mustard oils and their NEs (mean of both seasons).

Treatments				Nitrogen %	Protein %	TSS (mg/g DW)	TFAA (mg/g DW)	TPC (mg/g G.A DW)	TFC (mg/g Q DW)
Control	Faba bean only			4.40a	26.50b	42.73c	8.42a	2.32ab	2.73abc
	Unweeded			2.40g	15.00j	16.85n	2.78i	1.52e	1.66d
Rocket oil	Without NE	%	2.5	2.73f	23.50g	28.29j	5.95ef	2.08bc	2.52bc
			5.0	4.08b	25.00e	33.10g	7.36c	2.16bc	2.58abc
			7.5	4.44a	27.50a	48.03a	8.65a	2.48a	2.85a
	With NE		2.5	4.00bc	25.00e	32.57h	6.45d	2.16bc	2.57abc
			5.0	4.40a	26.50b	39.60d	7.80b	2.26ab	2.72abc
			7.5	3.56de	22.25 h	26.37l	5.57g	1.77de	2.45c
Mustard oil	Without NE	%	2.5	3.75cd	23.44g	26.47l	5.89f	1.93cd	2.47c
			5.0	3.75cd	25.50d	34.59f	7.50c	2.24ab	2.58abc
			7.5	4.42a	27.50a	45.93b	8.65a	2.46a	2.79ab
	With NE		2.5	4.16ab	24.25f	30.34i	6.21de	2.09bc	2.53bc
			5.0	4.16ab	26.00c	38.22e	7.53c	2.24ab	2.71abc
			7.5	3.36e	21.00i	22.80 m	5.22 h	1.74de	2.46c
P-value				**	**	**	**	**	**
SE				± 0.06	± 0.06	± 0.06	± 0.05	± 0.05	± 0.06

** Significative at a level of 1% of probability (*p* < .01), * Significative at a level of 5% of probability (0.01 = < *p* < .05), ns Non significative (p > = 0.05), NE = nano-emulsion, TSS = total soluble sugar, TFAA = total fatty amino acids, TPC = total phenolic compounds, TFC = total flavonoid compounds and SE = standard error.

## Discussion

As there are many problems attributed with chemical weed management. Weed scientists reward their efforts to find ecofriendly alternatives that have no side effects on human, animals and surrounding environment^36^. In this regard, the present study achieved the hypothesis of the ability to apply rocket and mustard oils and their NEs as natural bioherbicides for controlling canary grass and cheeseweed associated faba bean plants under greenhouse conditions. Moreover, these natural products achieved positive physiological response of faba bean plants that in turn reflected on yield through the inhibition of biotic stress (both investigated weeds).

Our fractionation to both rocket and mustard oils is in agreement with Gulfraz et al.^37^ who founded that the main compounds in salad rocket oil was erucic acid (51.2%) followed by oleic acid (15.15%) and cis11-eicosenoic acid (12.5%). Also, Koubaa et al.^38^ reported that linolenic acid was more pronounced in salad rocket oil with 30% of the total fatty acids while the major fatty acid in mustard oil is erucic acid being 35% of the total fatty acids. Additionally, Nail et al.^39^ recorded that GC-MS analysis of rocket oil showed 28 components as Palmitic acid (hexadecanoic acid), Stearic acid (octadecanoic acid), Oleic acid (Octadecenoic acid), Linoleic acid as (Octadecadienoic acid), Erucic acid (Docosenoic acid).

The characteristics of NE such as uniformity, dispensability, and stability were confirmed by particle size distribution, average particle size and PDI and TEM images^40^. As our results ensured that PDI values of rocket and mustard NE was 0.56 and 0.01, respectively and the droplet’s PDI value, which ranges from 0.08 to 0.70 and averages 0.38, guarantees a uniform distribution of droplet sizes and demonstrates to the translucent or transparent character of NE, that Danaei et al.^41^ stated that a PDI value larger than 0.7 denotes an extremely wide and heterogeneous range of droplet size. Zeta potentials below ± 10 are regarded to be significant because to their instability, whereas values above this range cause the formulation stable^42^. So, rocket and mustard oil NEs were stable due to its measured zeta potential of -33.56 and − 29.88 mV. The free fatty acids and other polar constituents in the oil phase may be adsorbed on the surface of the emulsion to be the source of the rocket and mustard oil NE’s negative charge^43^. TEM image proved that droplets had sphere-like shapes ranged from 47 to 65 for rocket oil and 53.3–76.9 for mustard oil. It was proven that NE formulated by Tween 20 & 80 and SDS was stable and oil in water (O/W) NEs. Collectively, nano-emulsified oils that reduced droplet size act through multiple mechanisms ranging from inhibition of germination through restriction of water uptake, α-amylase activity or enhancing the bioherbicidal activity by improving stability, penetration and delivery of active compounds to plant tissues through seed coats or leaf^44^.Recent studies revealed that nanoformulations of bioherbicides enable achieving the desired efficacy with minimum effective concentration of the active ingredients^45^.

Concerning to the bioherbicidal activity of both investigated oils, the application of fixed oils is limited, especially, rocket salad or mustard fixed oils as natural bioherbicides for controlling weeds. But some researchers agreed with our results by reporting that either rocket salad or mustard plants or its seed powder has allelopathic potentials to be applied as alternative natural herbicides. Messiha et al.^46^ concluded that rocket salad seed powder can be used as a selective bio-herbicide to control the perennial troublesome weed *Cyperus rotundus* infesting maize plants in the summer season. Whereas, El-Dabaa et al.^47^ revealed the efficiency of rocket salad seed powder to control parasitic broomrape that infecting faba bean plants. Moreover, El-Wakeel et al.^48^ indicated that fresh shoot alcoholic extract of rocket salad (foliar spray) reduced growth of canary grass weeds. Mixing of mustard meal powder with soil released allelopathic compounds had herbicidal effect on weeds^49,50^. Additionally, foliar spray of mustard seed extract acted as natural herbicide that may be attributed with glucosinolates and phenolic compounds^51^.

Bearing in mind that the reduction happened in weed growth parameters by the application of investigated oils or their NEs may be attributed with phytochemicals and bioactive compounds in rocket and mustard seed oil. According to Koubaa et al.^38^ polyphenol compounds such as sinapic acid, syringic acid, apigenin, ascorbic acid and vanillin were identified in rocket seed oil by HPLC-HRMS. Purkait et al.^52^ founded that GC-MS analysis of mustard oil revealed the presence of some phytochemicals such as 2-Decenal, (Z); 2,4-Deca- dienal, (E, E); 2-Undecanal; trans-13-Octadecenoic acid; Oleic acid, 3-(octadecyloxy) propyl ester. Some bioactive constituents were also determined in mustard oil i.e. Sitosterol; Campesterol; 6-Methyloctadecane; 17-Octadecynoic acid; 8-Heptadecene; 9-Hexadecenoic acid; Octadecane, 6-methyl; trans-13-Octadecenoic acid, methyl ester; cis-Vaccenic acid; Olein, 2 mono; cis-13-Octadecenoic acid; Glycidyl oleate; Ethyl iso-allocholate. However, results of Qanash et al.^53^ detected that GC-MS analysis of rocket oil proved the presence of palmitic acid, TMS derivative (44.7%) as the main compound and 3,5-di-t-butyl-4-hydroxybenzoic acid. Batista et al.^54^ concluded that fatty acids such as linolenic, linoleic, oleic and palmitic acids had a phytotoxic herbicidal effect on weeds and this phytotoxicity attributed with concentration and number of saturation of these fatty acids. Accordingly, rocket and mustard oils are composed of a wide spectrum of biologically active constituents that may act synergistically, additively, or antagonistically, making the elucidation of their exact mechanism of action highly challenging due to their complexity and chemical variability^55^. Batish et al.^56^ revealed that the application of essential oils led to significant reductions in chlorophyll content and cellular respiration of treated weeds, thereby negatively affecting photosynthetic efficiency and energy metabolism. The visible phytotoxic symptoms induced by essential oils exposure, including growth inhibition, chlorosis and leaf necrosis, have been attributed to their interference with critical physiological and cellular processes, such as the inhibition of mitosis, suppression of chlorophyll biosynthesis, disruption of mitochondrial respiration, membrane depolarization, ion leakage and removal of cuticular waxes^57^. Asadi et al. ^58^ ensured that fatty acids derived from glyceric plant oils can be applied as alternatives to chemical herbicides. Additionally, glucosinolates from Brassicaceae are enzymatically converted upon tissue disruption into bioactive isothiocyanates that rapidly compromise weed cell membranes, induce oxidative stress, and inhibit photosynthesis and respiration that subsequently suppressing weed growth^59^.

As rocket and mustard oils and their NEs reduced the dry weight of both weeds and consequently increased all faba bean growth parameters at vegetative and flowering stages through decreasing competition on all natural resources required for growth. This is in agreement with Mishra and Singh^60^, Meena et al.^61^ and El-Wakeel et al.^62^ who stated that the reduced competition between the crop and weeds during the vital period of crop growth may be the cause of the growth improvement. There are few publications ensure growth-stimulating effect of crude extracted plant fixed oils. It has been reported that either crude oil or fatty acids encourage the production of citric acids, carotenes and enzymes^63^. Yang et al.^64^ ensured that plant oils have been recorded as accelerator to polysaccharides and mycelia in some mushroom species. Additionally, the carbon chain length and concentration of fatty acid determine its stimulation or inhibition activity. Meanly, palmitic and oleic acids are reported as great accelerators to polysaccharide production and mycelial growth. In contrast, linoleic acid suppressed polysaccharide formation that in turn inhibited mycelial growth. Hence, the growth stimulation of faba bean plants may be attributed with palmitic and oleic acids that have been identified in abundant quantity in both oils. Park et al.^65^ concluded that the crude plant fixed oils were active recommended growth regulators than the sole application of fatty acids.

Photosynthesis depends on plant pigments like chlorophylls a and b as well as carotenoids. One of the most significant biochemical characteristics that can reveal a plant’s state of health is chlorophyll. Its concentration is correlated with plant nutrient uptake and water accessibility. However, chlorophyll b is the vital regulator of the biosynthesis in photosynthetic process as it considered as a main source of signals for plant growth and development^66^. The current investigation showed that canary grass and cheeseweed reduced leaf chlorophyll content and decreasing Photosynthesis efficiency. Vice versa, all treatments at different concentrations improved and increased photosynthetic pigment levels (chlorophyll a, chlorophyll b & carotenoids) at vegetative and flowering stages may be related to the improvement of pigment production or a delay in pigment degradation^67^. Dyadiuchenko et al.^68^ have been approved anise, fenchel and apricot oils as friendly growth regulators for wheat yield through stimulation of chlorophyll content in wheat leaves.

Weeds cause great losses in seed yield of bean because of direct competition between weeds and plants for light, moisture and soil nutrients. Mekky and Atwa^5^ reported that weed competition cause losing faba bean yield (exceed 50%). Our results revealed that the reduction of investigated weeds positively reflected in turn on faba bean growth parameters, photosynthetic pigments and yield traits. According to several researchers, weed control reduced weeds’ ability to compete with crop plants, which reduced interference with crop plants and increased net income in plant growth and yield^57,69,70^. Increased seed yield refers to the capacity of plant species to transform as much of the result of photosynthesis into seeds as possible depending on the quantity of the supplied assimilates and the seed capacity to store those^71^.

Faba bean seeds quality increased due to the application of rocket and mustard oils and their NEs as compared to unweeded control. This may be attributed with the reduction of biotic stress of infectious weeds which consequently caused significant increases in growth parameters, photosynthetic pigments, seed yield and its components. These results in agreement with El-Metwally et al.^72^ who stated that two hand hoeing treatment increased expressively total carbohydrate and protein percentages compared to controls. Therefore, the competition was restricted thus light, water and nutrients are being available to faba bean growth. Stimulation of Rubisco activity and improvement of photosynthetic pigments lead to increasing of soluble sugars^73^.

## Conclusions

Rocket and mustard oils and their NEs achieved our hypothesis in having herbicidal properties as they have high phytotoxic effect on growth and physiological processes of the weeds. The management of weeds is reflected on faba bean growth and physiological parameters as well as yield traits. So, Rocket and Mustard oils NEs can be recommended as eco-friendly natural herbicides for controlling two weeds under investigation (canary grass and cheeseweed) infected faba bean plants in organic agricultural systems.

### Future work

Applying rocket and mustard oil NEs as natural bioherbicides to control canary grass and cheeseweed under the field level to achieve organic production of faba bean crop. Testing the efficiency of rocket and mustard oil NEs on different types of weeds with different growing crops.

## Data Availability

The data used for this study are available upon request by contacting the corresponding author.

## Abbreviations

DAS: Days after sowing
PdI: Polydispersity index
NE: Nano-emulsion
FW: Fresh weight
DW: Dry weight
GC: Gas chromatography
TEM: Transmission electron microscope
FA: Fatty acid
TN: Total nitrogen
TP: Total protein
TSS: Total soluble sugar
TFAA: Total free amino acid
TPC: Total phenolic compounds
TFC: Total flavonoid content
